# Supplementary material for: Mitochondrial DNA removal is essential for sperm development and activity
Source: EMBO J. 2025 Feb 11;44(6):1749–73. doi: 10.1038/s44318-025-00377-5 (PMC11914152; doi:10.1038/s44318-025-00377-5)
Supplement: Supplementary file 1 — Appendix [file 44318_2025_377_MOESM1_ESM.pdf]

Appendix

Mitochondrial DNA removal is essential for sperm development and activity

Zhe Chen et al.

* Correspondence: Hong Xu (hong.xu@nih.gov)

Contents

Appendix Figure S1 2
Appendix Figure S2 3
Appendix Figure S3 4

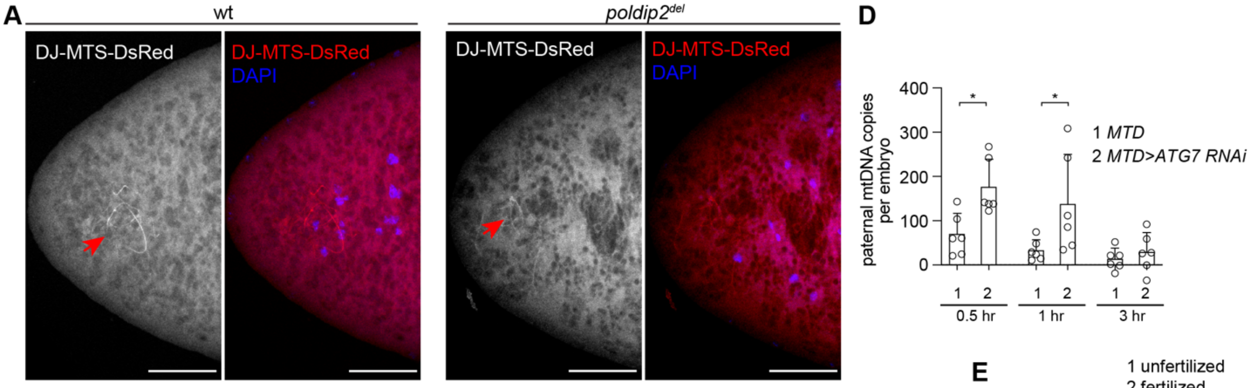

B

% embryos	wt (n=48) non-developing	wt (n=48) developing	*poldip2*^{del} (n=54) non-developing	*poldip2*^{del} (n=54) developing
sperm observed	0	64.6	0	12.9
sperm not observed	14.6	20.8	81.5	5.6

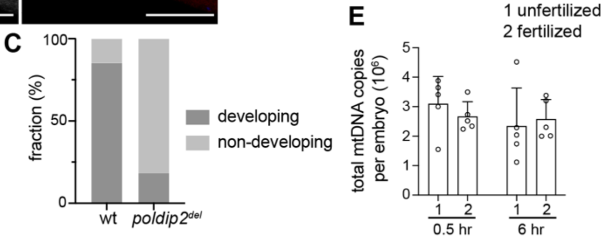

Appendix Fig. S1. (**A**) Representative images showing sperm in early embryos. Male wt or *poldip2*^{del} flies carrying DJ-MTS-Red transgene were crossed with *w*^{1118} female flies. Embryos were collected 0-30 min post-laying and analyzed. Anterior of the embryo is oriented to the left. DJ-MTS-Red labels the mitochondrial derivative of the sperm tail (arrows). DAPI stains nuclear DNA. Embryos with multiple nuclei indicate ongoing development. Bar, 50 μm. (**B, C**) Quantification of the proportion of developing embryos (DAPI staining) and embryos with detectable sperm (labeled by DJ-MTS-Red) fertilized by wt and *poldip2*^{del} sperm. Notably, no non-developing embryos contained sperm in either group, suggesting that both wt and *poldip2*^{del} sperm can initiate embryonic development. Sperm were not observed in a subset of developing embryos, potentially due to low DJ-MTS-Red signal intensity that could not be distinguished from background autofluorescence. (**D**) Droplet digital PCR (ddPCR) quantification of *poldip2*^{del} sperm-derived mtDNA in ATG7 knockdown embryos. Crosses were performed between female *MTD-gal4>ATG7* RNAi (*mt:ND2*^{del1}) and male *poldip2*^{del} (*mt:wt*) flies. Embryos were collected 0-30 min post-laying and analyzed immediately (0.5 h), or after 1h or 3 h of development. Crosses between female *MTD-gal4* (*mt:ND2*^{del1}) and male *poldip2*^{del} (*mt:wt*) were used as controls. Each data point represents a biological replicate (n=6). The data represent the mean± SD. Statistical analysis was performed using an unpaired *t*-test. * P<0.05. (**E**) Droplet digital PCR (ddPCR) quantification of total mtDNA in embryos using primers/probe targeting mtDNA-encoded *mt:CoI*. Embryos were collected from virgin or mated *w*^{1118} (*mt:ND2*^{del1}) female flies 0-30 min post-laying and analyzed immediately (0.5 h) or after 6 h. Each data point represents a biological replicate (n=5). The data represent the mean± SD.

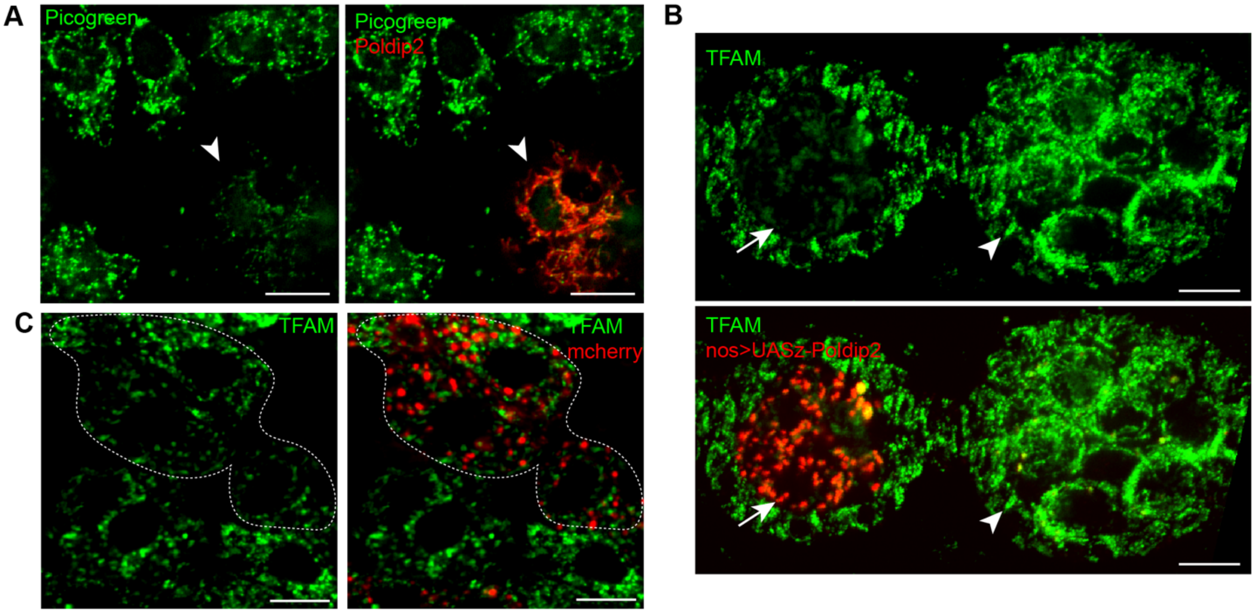

Appendix Fig. S2. (**A**) Representative image showing that Poldip2 overexpression in S2 cells reduces mtDNA level, as indicated by reduced Picogreen staining. Note that cells expressing Poldip2 (arrowhead) show lower Picogreen intensity compared to neighboring cells lacking Poldip2 expression. Bar, 10 μm. (**B**) Poldip2 ectopically expressed in ovarian germ cells driven by *nanos-gal4* reduced mtDNA levels labeled by TFAM-mNeonGreen. The egg chamber with high Poldip2 expression display decreased TFAM intensity in germ cells (arrows). In contrast, the egg chamber with negligible Poldip2 expression show comparable TFAM levels between germ cells and surrounding follicle cells (arrowheads). Bar, 10 μm. (**C**) Representative image of Poldip2 overexpression in midgut enterocyte clones. Poldip2-expressing clones are labeled with mCherry (red) and exhibit variable mtDNA levels, as indicated by TFAM, when compared to non-expressing neighboring cells. The dashed white line outlines the clonal cells. Bar, 10 μm.

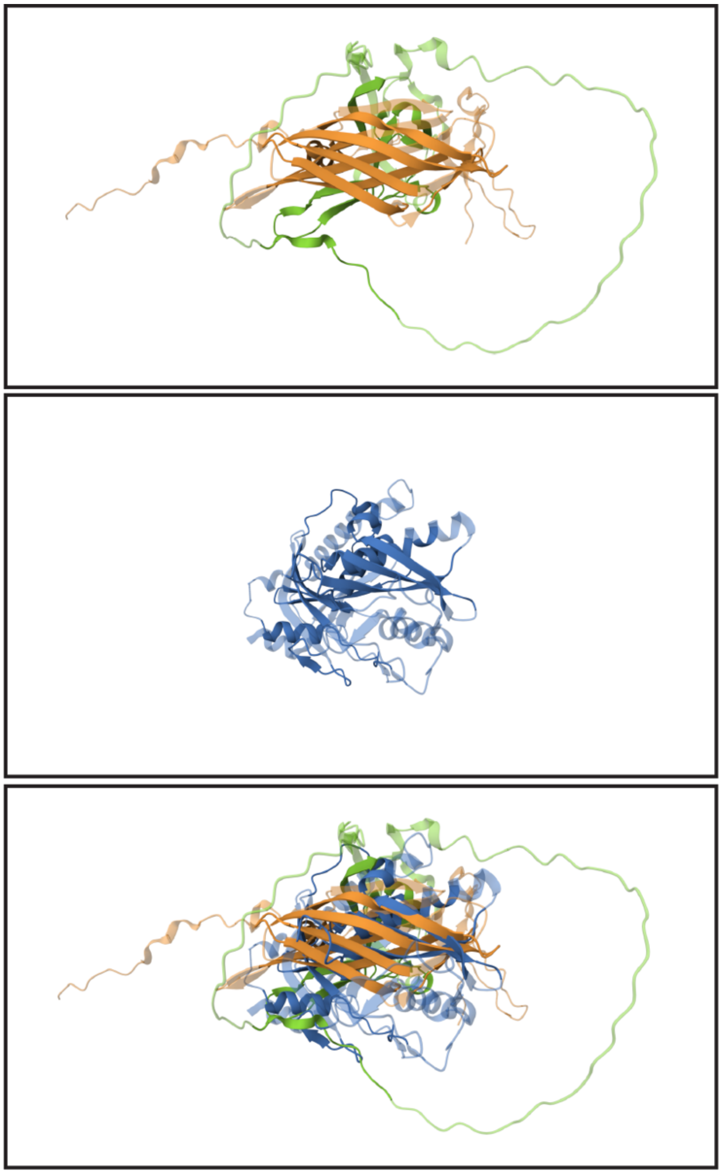

Appendix Fig. S3. Comparison of the AlphaFold-predicted model of *Drosophila* Poldip2 (Alphafold DB: Q9VNC0) with the crystal structure of mammalian Dom3Z (PDB: 3FQJ), an RNA nuclease with 5′-3′ exoribonuclease activity. The *Drosophila* Poldip2 YccV domain (green) and ApaG domain (orange) are labeled according to InterPro. The C-terminal ApaG domain (orange) of Poldip2 displays moderate structural similarity to Dom3Z (blue) with the TM score of 0.21.
