# Supplementary figures and images for: Mitochondrial DNA removal is essential for sperm development and activity
Source: EMBO J. 2025 Feb 11;44(6):1749–73. doi: 10.1038/s44318-025-00377-5 (PMC11914152; doi:10.1038/s44318-025-00377-5)

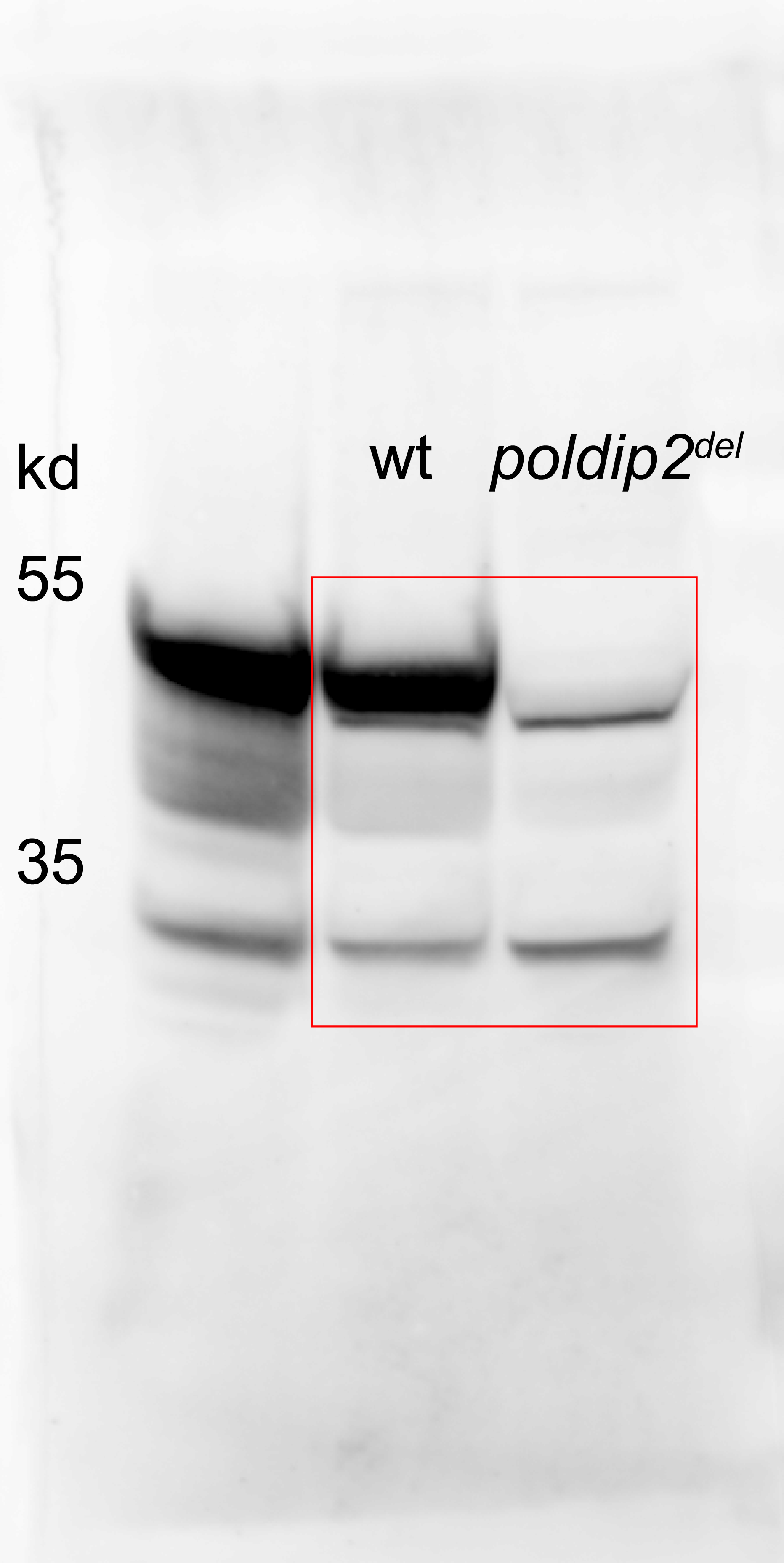

Supplement: Supplementary file 7 — Source data Fig. 1 [file 44318_2025_377_MOESM7_ESM.zip › Figure 1/1D/1D_Western Poldip2.tif]
