# Supplementary material for: Mitochondrial DNA removal is essential for sperm development and activity
Source: EMBO J. 2025 Feb 11;44(6):1749–73. doi: 10.1038/s44318-025-00377-5 (PMC11914152; doi:10.1038/s44318-025-00377-5)
Supplement: Supplementary file 12 — Expanded View Figures [file 44318_2025_377_MOESM12_ESM.pdf]

Expanded View Figures

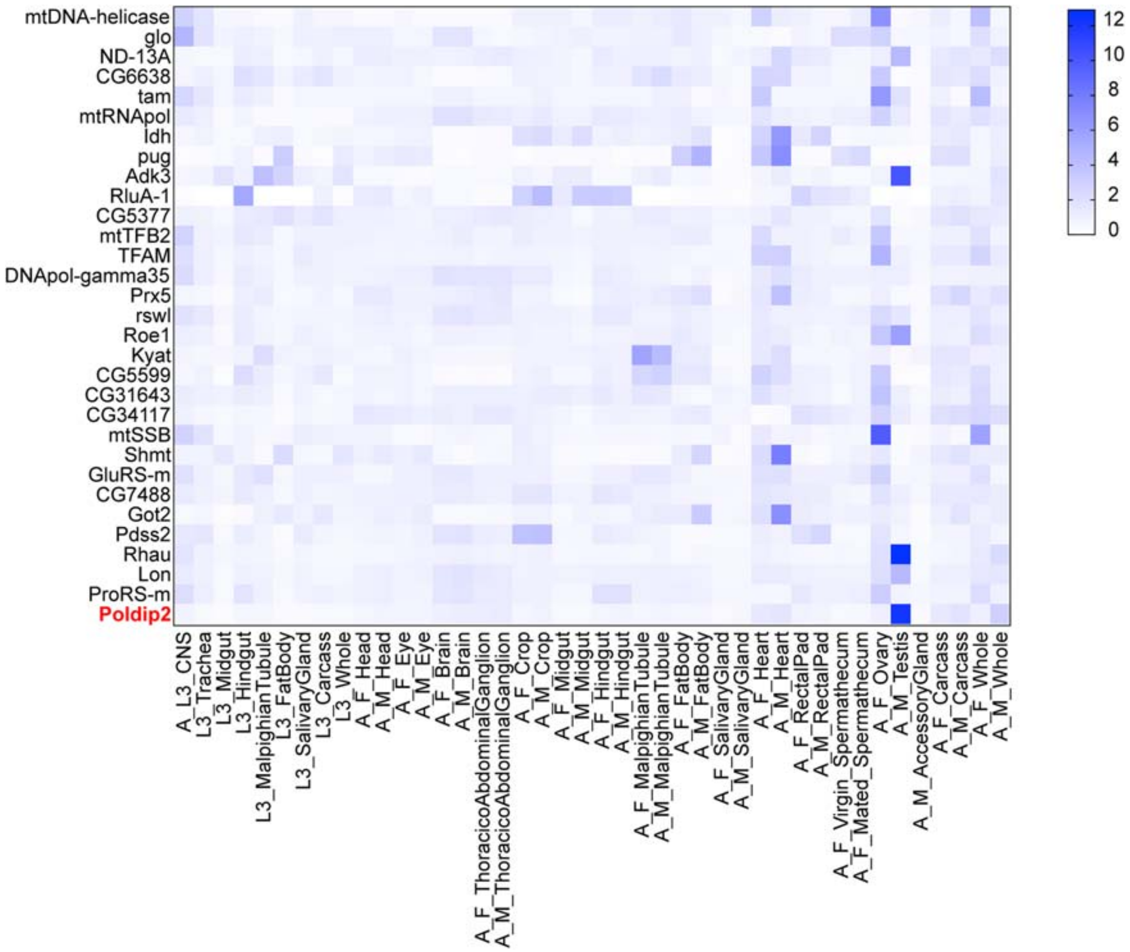

Figure EV1. Tissue expression profile of *Drosophila* mitochondrial nucleoid-associated proteins.

The heatmap was generated with RNA-seq data from FlyAtlas2 (the *Drosophila* gene expression atlas). Color codes indicate the scaled RPKM (reads per kilobase per million mapped reads) folds over tissues. Note that the mRNA level of Poldip2 is significantly higher in *Drosophila* testes compared to other tissues. A, adult; L3, third instar larva; F, female; M, male.

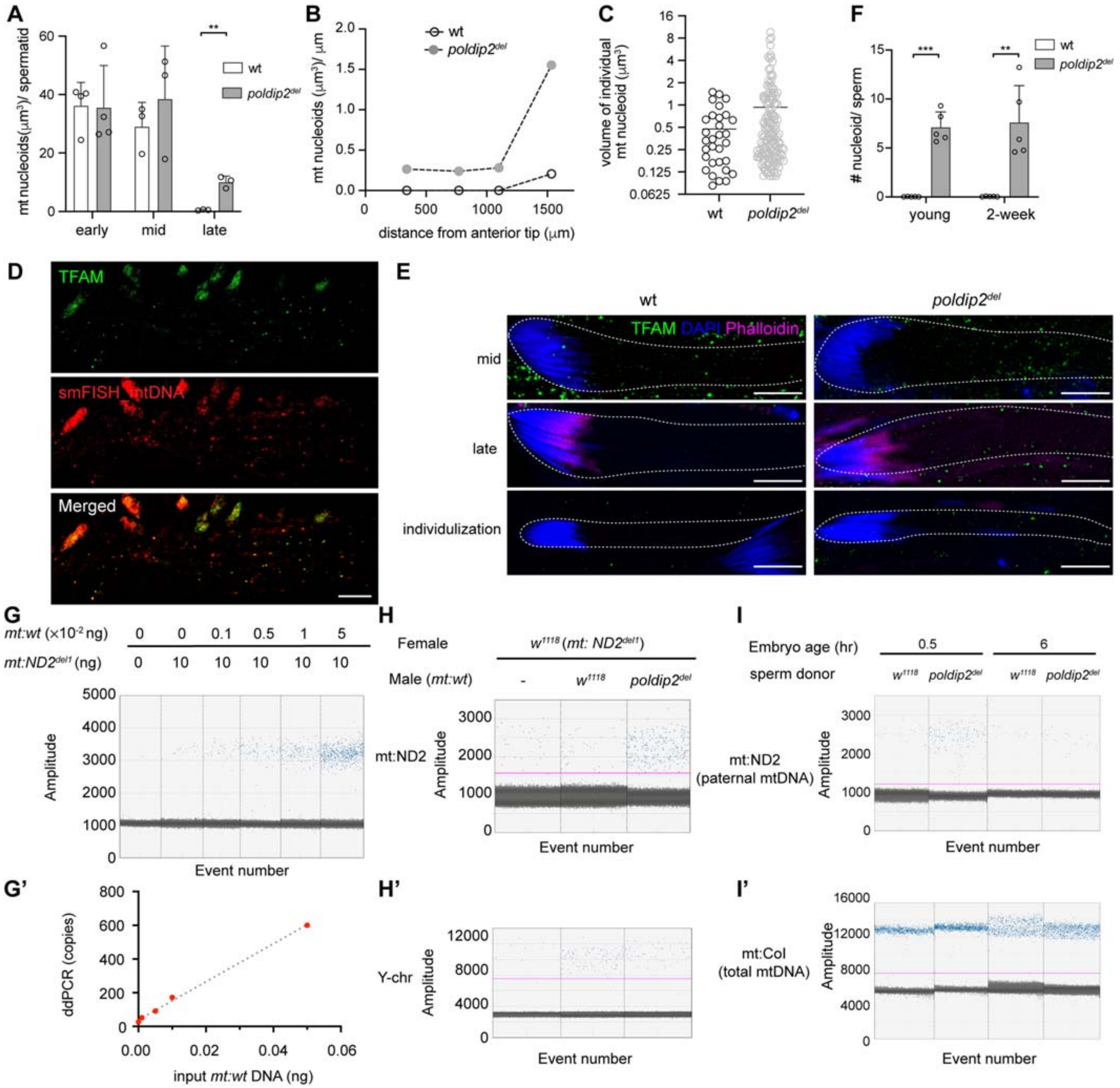
A
mt nucleoids(μm³)/ spermatid
wt
poldip2del
early
mid
late
**
B
mt nucleoids (μm³)/ μm
distance from anterior tip (μm)
C
volume of individual mt nucleoid (μm³)
wt
poldip2del
F
nucleoid/ sperm
young
2-week
***
**
D
TFAM
smFISH_mtDNA
Merged
E
TFAM DAPI Phalloidin
mid
late
individulization
G
mt:wt (×10⁻² ng)
mt:ND2del1 (ng)
Amplitude
Event number
G'
ddPCR (copies)
input mt:wt DNA (ng)
H
Female
w1118 (mt: ND2del1)
Male (mt:wt)
mt:ND2
H'
Y-chr
I
Embryo age (hr)
sperm donor
mt:ND2 (paternal mtDNA)
I'
mt:CoI (total mtDNA)

Figure EV2. Mitochondrial DNA persists in late spermatogenesis stages and mature sperm of *poldip2^{del}* flies.

(**A**) Total mitochondrial nucleoids measured in volumes per spermatid at early-elongating (early), mid-elongating (mid) and fully elongated (late) stages. Each data point represents a spermatid ($n = 3, 4$). The data represent the mean± SD. Statistical analysis was performed using an unpaired *t* test. **$P = 0.0012$. (**B**) The density of mitochondrial nucleoids (total volumes per µm) along the length of a representative fully elongated spermatid bundle for w^{1118} (wt) and *poldip2^{del}* flies, respectively. (**C**) A scatter dot plot displaying the individual mitochondrial nucleoid volumes from the fully elongated spermatids of w^{1118} (wt) and *poldip2^{del}* flies. Each data point represents an individual nucleoid (wt, $n = 31$; *poldip2^{del}*, $n = 153$). The solid lines indicate the mean volume. (**D**) TFAM-mNeonGreen can be used as a mitochondrial nucleoid marker in *Drosophila* testis. Single-molecule fluorescent in situ hybridization (smFISH) signals using fluorescently labeled DNA probes specific for mtDNA (red), are colocalized with TFAM-mNeonGreen in *Drosophila* testis. Bar, 10 µm. (**E**) Mitochondrial nucleoids labeled by TFAM-mNeonGreen demonstrate a consistent pattern of mtDNA elimination during spermatogenesis in both w^{1118} (wt) and *poldip2^{del}* flies, compared with DNA dye (DAPI) staining. In elongating spermatids of both wt and *poldip2^{del}* testis, intense TFAM-mNeonGreen puncta signals were detected. The signals were rare in fully elongated and individualization stage spermatids of wt flies. Conversely, persistent mtDNA was frequently observed in the same stages of *poldip2^{del}* spermatids. Phalloidin (magenta) stains actin; DAPI (blue) stains nuDNA. Bar, 10 µm. (**F**) Quantification of mitochondrial nucleoid numbers in mature sperm of young and 2-week-old flies. Each data point represents quantification from one seminal vesicle ($n = 5$). The data represent the mean± SD. Statistical analysis was performed using an unpaired *t* test. ***$P = 7.0 \times 10^{-6}$, **$P = 0.0019$. (**G**) Evaluating the specificity of the primers/probe set targeting mtDNA-encoded ND2 locus (*mt:ND2*) using droplet digital PCR (ddPCR) assay. A reaction containing 10 ng of total DNA from w^{1118} (*mt:ND2^{del1}*), a fly strain carrying a 9-base pair deletion on the mtDNA-encoded ND2 locus, mixed with 0, 0.001, 0.005, 0.01 or 0.05 ng of total DNA from w^{1118} (*mt:wt*, wild-type mtDNA) flies, was performed. The ddPCR primers/probe were designed to target the *mt:wt* while excluding *mt:ND2^{del1}* mtDNA. (**G'**) Correlation of the amount of input w^{1118} (*mt:wt*) DNA with the resulting mtDNA copy numbers using ddPCR. Simple linear regression was carried out and the coefficient of correlation $R^2 = 0.9947$. (**H–H'**) Quantification of mtDNA copy numbers per sperm in w^{1118} and *poldip2^{del}* flies using ddPCR. Crosses were conducted between female w^{1118} (*mt:ND2^{del1}*) and male w^{1118} (*mt:wt*) or *poldip2^{del}* (*mt:wt*) flies. Then the total DNA from the female spermatheca was extracted and analyzed. Virgin female w^{1118} (*mt:ND2^{del1}*) flies were used as the negative control. The primers/probe sets were designed to target *mt:ND2* (**H**) and Y-chromosome gene *kl-2* (**H'**), respectively. The input total DNA for detecting *mt:ND2* gene is 5 ng for each reaction. The input total DNA for detecting the Y-chromosome gene is 125 ng for each reaction. (**I–I'**) Analysis of sperm-derived mtDNA in embryos using ddPCR. Crosses were performed between female w^{1118} (*mt:ND2^{del1}*) and male w^{1118} (*mt:wt*) or *poldip2^{del}* (*mt:wt*) flies. Embryos were collected 0–30 min post-laying and analyzed immediately (0.5 h) or after 6 h (6 h) of development. Primers/probe sets targeting *mt:ND2* (**I**) and *mt:CoI* (**I'**) were used to quantify paternal mtDNA and total mtDNA, respectively. Total mtDNA levels in embryos remain constant up to 10 h after egg-laying (Rubenstein et al, 1977), averaging 2.5×10^6 copies per embryo (Appendix Fig. S1E). Input maternal mtDNA for detecting *mt:ND2* and *mt:CoI* was 2.5×10^6 and 2×10^3 copies per reaction, respectively.

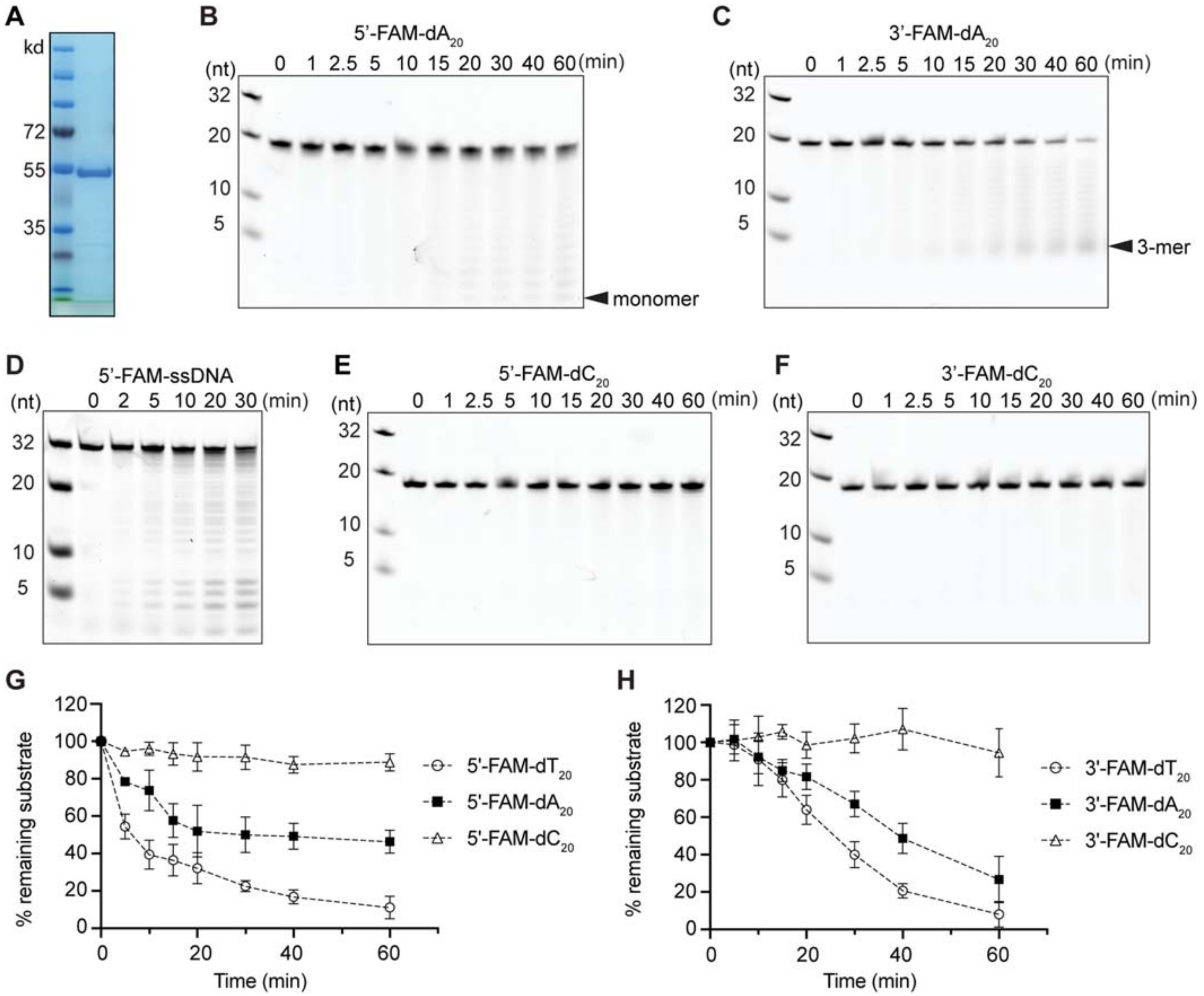

Figure EV3. Poldip2 is a mitochondrial DNA exonuclease.

(**A**) SDS-PAGE of the purified Poldip2 protein. M, molecular weight marker. (**B**, **C**) Degradation pattern of 5′-6-FAM and 3′-6-FAM-labeled 20-nt poly (dA) single-stranded (ssDNA) substrates. The 100 nM 5′-6-FAM (**B**) or 3′-6-FAM (**C**) labeled 20-nt poly (dA) was incubated with Poldip2 protein (200 nM) at 37 °C and analyzed at the indicated time points. (**D**) Degradation pattern of a 5′-6-FAM-labeled ssDNA substrate consisting of mixed dA, dT, dC and dG. The 100 nM 5′-6-FAM-labeled 32-nt ssDNA was incubated with Poldip2 protein (200 nM) at 37 °C and analyzed at the indicated time points. (**E**, **F**) Degradation pattern of 5′-6-FAM and 3′-6-FAM-labeled 20-nt poly (dC) ssDNA substrates. The 100 nM 5′-6-FAM (**E**) or 3′-6-FAM (**F**) labeled 20-nt poly(dC) was incubated with Poldip2 protein (200 nM) at 37 °C and analyzed at the indicated time points. (**G**, **H**) Quantification of the remaining full-length substrates, including 5′-6-FAM (**G**) or 3′-6-FAM (**H**) labeled 20-nt poly (dT), poly (dA) and poly (dC), at each time point. Data are normalized to the initial level of the full-length substrates and plotted ($n = 3$). The molecular markers in this figure are an equal molar mixture of 5′-6-FAM-labeled 32-nt, 20-nt, 10-nt and 5-nt oligonucleotides and were loaded at a concentration of 50 nM for each. The data represent the mean± SD.

 © The Author(s)

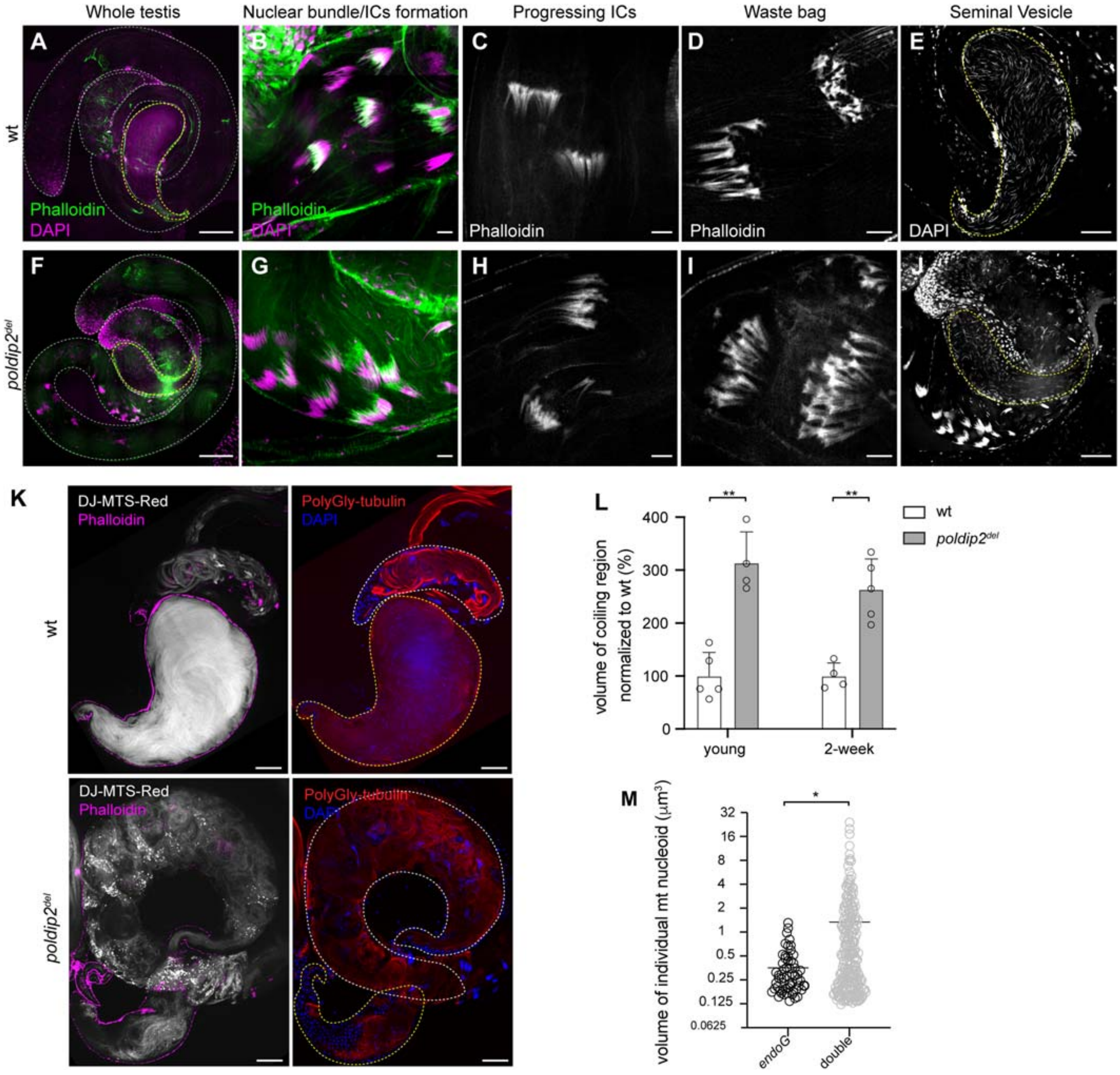

Figure EV4. Persistent mtDNA impedes spermatid individualization.

(**A–J**) Representative images showing the whole testis (**A**, **F**), individualization complexes (ICs) formation next to the nuclear head in the testis basal region (**B**, **G**), actin cone structures in progressing ICs (**C**, **H**), waste bags (**D**, **I**), and seminal vesicles (**E**, **J**, yellow dashed line) in w^{1118} (wt) and *poldip2*^{del} flies. Phalloidin stains actin; DAPI stains nuDNA. Bar, 100 μm in (**A**) and (**F**); 10 μm in (**B–D**) and (**G–I**); 50 μm in (**E**) and (**J**). (**K**) Representative images showing the coiling region (white dashed line) and seminal vesicle (yellow dashed line) of w^{1118} (wt) and *poldip2*^{del} flies. DJ-MTS-Red stains the mitochondria derivatives; Polyglycylated tubulin stains fully elongated axonemal microtubules; Phalloidin stains actin; DAPI stains nuDNA. Bar, 50 μm. (**L**) The coiling region in both young and 2-week-old *poldip2*^{del} flies is enlarged compared to wt control. Each data point represents quantification from one testis ($n = 4, 5$). The data represent the mean± SD. Statistical analysis was performed using an unpaired t test. P values from left to right: $^{**}P = 0.00042$, $P = 0.0012$. (**M**) A scatter dot plot displaying the individual mitochondrial nucleoid volumes from the elongated spermatids of *endoG* (*endoG*$^{MB07150/KO}$) and double mutants (*endoG*$^{MB07150/KO}$; *poldip2*^{del}). Each data point represents an individual nucleoid (*endoG*, $n = 58$; double mutant, $n = 248$). The solid lines indicate the mean volume. Statistical analysis was performed using an unpaired t test. $^{*}P = 0.0058$.

© The Author(s)

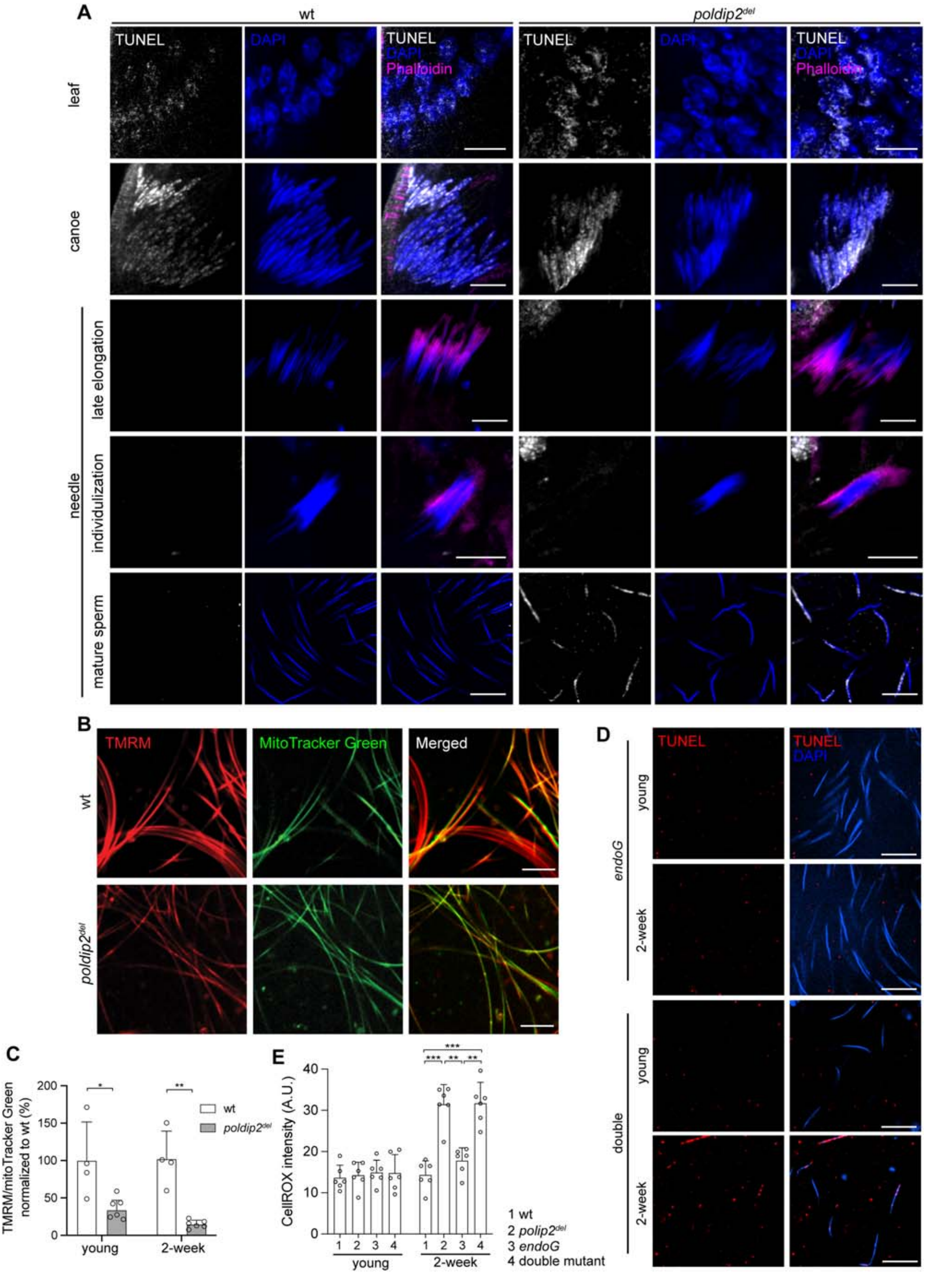
A
wt
poldip2del
TUNEL
DAPI
TUNEL DAPI Phalloidin
leaf
canoe
late elongation
individulization
mature sperm
needle
B
TMRM
MitoTracker Green
Merged
wt
poldip2del
C
TMRM/mitoTracker Green normalized to wt (%)
wt
poldip2del
young
2-week
E
CellROX intensity (A.U.)
1 wt
2 polip2del
3 endoG
4 double mutant
young
2-week
D
TUNEL
TUNEL DAPI
endoG
double
young
2-week

 © The Author(s)

Figure EV5. Persistent mtDNA in mature sperm causes nuclear DNA fragmentation.

(**A**) Representative images showing nuDNA breaks labeled by TUNEL assay in the process of chromatin remodeling during spermatogenesis. The highest abundance of TUNEL signal (white) was observed in the late canoe stage, which corresponds to the histone-to-protamine transition phase. The nuDNA breaks subsequently disappeared in needle-shaped nuclei during late elongation and individualization stages, indicating the repair of nuDNA breaks after the transition. No significant differences were observed between wt and *poldip2*^{del} flies throughout this process. The developmental stages were distinguished by the morphology of nuclear heads stained with DAPI (blue), and the positioning of actin cones stained with Phalloidin (magenta). Bar, 10 µm. (**B**) Compromised mitochondrial membrane potential of *poldip2*^{del} sperm. Mature sperm from *w*^{1118} (wt) and *poldip2*^{del} seminal vesicles were stained with TMRM (red), a dye sensitive to mitochondrial membrane potential, in combination with MitoTracker Green (green) as a reference. Bar, 10 µm. (**C**) Quantification of TMRM/MitoTracker Green ratios in both young and 2-week-old flies. Each data point represents quantification from one seminal vesicle ($n = 4, 6$). The data represent the mean± SD. Statistical analysis was performed using an unpaired *t* test. *$P = 0.019$, **$P = 0.00039$. (**D**) Representative images of TUNEL assay in *endoG* (*endoG*$^{MB07150/KO}$) and double mutant (*endoG*$^{MB07150/KO}$; *poldip2*^{del}) seminal vesicles. The nuDNA breaks/fragmentation was observed in 2-week-old double mutant mature sperm. DAPI (blue) stains the nuDNA of mature sperm. Red: TUNEL signal. Bar, 10 µm.
(E) Quantification of CellROX intensity, as a measure of ROS levels in both young and 2-week-old flies with indicated genotypes. (1) *endoG*^{MB07150}/+; *poldip2* ^{del}/+. (2) *endoG*^{MB07150}/+; *poldip2* ^{del}. (3) *endoG*$^{MB07150/KO}$; *poldip2* ^{del}/+. (4) *endoG*$^{MB07150/KO}$; *poldip2*^{del}. A.U., arbitrary unit. Each data point represents quantification from one seminal vesicle ($n = 6$). The data represent the mean± SD. Statistical analysis was performed using an unpaired *t* test. *P* values from left to right: ***$P = 0.000038$, ***$P = 0.000032$, **$P = 0.00016$, **$P = 0.00018$.

© The Author(s)
